# RETRACTION: Impact of Perioperative Enhanced Recovery After Surgery on Wound Infection in Patients Undergoing Orthopaedic Surgery: A Meta‐Analysis

**DOI:** 10.1111/iwj.70283

**Published:** 2025-03-03

## Abstract

**RETRACTION:** NiuG.‐X. , LvA.‐H. , PengY.‐J. , and ZhangG.‐Q. , “Impact of Perioperative Enhanced Recovery After Surgery on Wound Infection in Patients Undergoing Orthopaedic Surgery: A Meta‐Analysis,” International Wound Journal 21, no. 4 (2024): e14555, 10.1111/iwj.14555.38158640 PMC10961859

The above article, published online on 29 December 2023, in Wiley Online Library (http://onlinelibrary.wiley.com/), has been retracted by agreement between the journal Editor in Chief, Professor Keith Harding; and John Wiley & Sons Ltd. Following an investigation by the publisher, all parties have concluded that this article was accepted solely on the basis of a compromised peer review process. The editors have therefore decided to retract the article. The authors did not respond to our notice regarding the retraction.

**RETRACTION:** G.‐X. Niu , A.‐H. Lv , Y.‐J. Peng , and G.‐Q. Zhang , “Impact of Perioperative Enhanced Recovery After Surgery on Wound Infection in Patients Undergoing Orthopaedic Surgery: A Meta‐Analysis,” International Wound Journal 21, no. 4 (2024): e14555, 10.1111/iwj.14555.38158640 PMC10961859

The above article, published online on 29 December 2023, in Wiley Online Library (http://onlinelibrary.wiley.com/), has been retracted by agreement between the journal Editor in Chief, Professor Keith Harding; and John Wiley & Sons Ltd. Following an investigation by the publisher, all parties have concluded that this article was accepted solely on the basis of a compromised peer review process. The editors have therefore decided to retract the article. The authors did not respond to our notice regarding the retraction.

